# Evaluating Primary Health Care Performance from User Perspective in China: Review of Survey Instruments and Implementation Issues

**DOI:** 10.3390/ijerph16060926

**Published:** 2019-03-14

**Authors:** Wenhua Wang, Jeannie Haggerty, Ekaterina (Katya) Loban, Xiaoyun Liu

**Affiliations:** 1 Department of Family Medicine, McGill University, Montreal, QC H3T 1M5, Canada; wenhua.wang@mail.mcgill.ca (W.W.); Jeannie.haggerty@mcgill.ca (J.H.); ekaterina.loban@mail.mcgill.ca (E.(K.)L.); 2 China Center for Health Development Studies, Peking University, Beijing 100191, China

**Keywords:** primary health care, performance evaluation, user experience, patient survey, China

## Abstract

This review aims to summarize the progress of patient evaluation studies focusing on primary health care (PHC) in China, specifically in relation to survey instruments and implementation issues. Eligible studies published in English or Chinese were obtained through online searches of PubMed and China National Knowledge Infrastructure. A descriptive reporting approach was used due to variations in the measurements and administration methods between studies. A total of 471 articles were identified and of these articles; of those 91 full-text articles were included in the final analysis. Most studies used author-developed measurements with five-point Likert response scales and many used the Chinese translations of validated tools from other countries. Most instruments assessed the physical environment, medical equipment, clinical competency and convenience aspects of PHC using a satisfaction rating instead of care experience reporting. Many studies did not report the sampling approach, patient recruitment procedures and survey administration modes. The patient exit survey was the most commonly used survey implementation method. The focus on the structural dimensions of PHC, inconsistent wording, categories of response options that use satisfaction rating, and unclear survey implementation processes are common problems in patient evaluation studies of PHC in China. Further studies are necessary to identify population preferences of PHC in China in order to move towards developing Chinese value-based patient experience measurements.

## 1. Introduction

In China, the primary health care (PHC) system set up in the 1950s has contributed to a dramatic reduction in maternal and infant mortality, a significant improvement in general population health and the development of the Declaration of Alma-Ata in 1978 [1]. However, the market economic reform, which began in 1978, undermined the previously established PHC system [2]. Instead, a hospital-centered fragmented delivery system was developed where health facilities at different levels were encouraged to self-finance through user charges and price mark-ups on medications and procedures. These developments incentivized high technology and low interinstitutional referral, with escalating medical costs, low affordability, public dissatisfaction and a significant disparity between urban and rural areas [3,4,5,6,7,8].

To address these issues, China launched a national health reform in 2009 with the aim of providing its citizens with essential public health and basic medical services that are safe, effective, accessible and affordable through a strong PHC based health system [9]. Guided by this national strategy, China has made a significant investment in health infrastructure at the PHC level and made significant progress in strengthening the primary care workforce. Combined with the impact of expanding social health insurance coverage, that provides a basic public health service package and an essential medicines program, China has witnessed a more equitable access to health care and greater affordability [1,4]. To deepen the health reform efforts, China is currently reshaping its tiered health care delivery system in accordance with the World Health Organization (WHO) Framework on Integrated People-Centered Health Services [10], which calls for health services to be organized around the health needs and preferences of individuals and requires active patient engagement in the care-seeking process [11]. There is growing interest in measuring PHC performance from a patient perspective in order to assess the Chinese reform effectiveness. However, the survey instruments and implementation approaches used vary significantly between studies.

Patient experience has been studied extensively in developed countries. It is recognized as one of the pillars of health care quality alongside clinical effectiveness and patient safety [12]. Patient experience during the service delivery process is also a major determinant of trust in and satisfaction with PHC [13]. Patients are the best evaluators of key aspects of PHC, such as interpersonal communication, shared decision-making, relational continuity, respect, privacy and advocacy [14]. With a positive care experience, patients are more likely to adhere to the recommended prevention methods and treatments, which subsequently results in better clinical and health outcomes [12,15,16]. Positive patient experience is also associated with a reduced use of health care resources and lower health care costs [17,18]. As an important indicator in health care performance evaluation, patient experience is included in quality improvement programs in developed countries, including the United Kingdom (UK), Australia, Canada and the United States of America (USA) [19]. 

Some of the widely used patient experience instruments in developed countries include the Primary Care Assessment Survey (PCAS) [20]; the Consumer Assessment of Healthcare Providers and Systems (CAHPS) [21,22]; the World Health Organization Health System Responsiveness Survey [23]; and the Primary Care Assessment Tools (PCAT) [24]. Several lessons have been learned from the development and application of these instruments. First, PHC domains and items should be selected based on health system features and population preferences in a specific context. For example, to adapt PCAS from the USA to the UK, several modifications were made, including rewording some questions, removing questions that are not relevant in the UK and adding domains relating to patient priorities in the UK [25]. Second, the wording of item response scales should reflect the response tendency of the local population. For example, for Canadians, Likert response scales function best and agreement scales are least appreciated [26]. Third, survey administration methods may influence the measurement error. For example, telephone respondents tend to give more positive responses than mail respondents [27]. 

These lessons provide practical information for patient survey design and implementation in developing countries, including China, where there is growing interest in measuring PHC performance from a user perspective. This literature review aims to summarize the progress and issues with patient evaluation studies of PHC in China in relation to three aspects: the choice of domains and items, the choice of response scales, and survey administration methods. The results will lay a solid foundation for further development of patient experience measurement methods in China. 

## 2. Methods

### 2.1. Databases and Search Terms

PubMed and China National Knowledge Infrastructure (CNKI) were searched to identify relevant articles. The PubMed and CNKI searches were conducted in February 2018. The following key search terms and their synonyms were used: China, primary health care, primary care, community health, township health center, village clinic, assessment, evaluation, measurement and patient. An iterative approach was used to identify search terms. The final search strategy can be found in Supplement 1. Only the publications in English and Chinese were retained. No time limit was imposed. 

### 2.2. Selection of Studies for Inclusion

A total of 169 articles in English and 302 articles in Chinese were identified. The following inclusion criteria were used: (1) the study is part of original research in China; (2) the study design is a patient survey; (3) the study reports the information on the measurement and administration of patient surveys. The screening process was conducted by one researcher. After screening the titles and abstracts, 37 articles in English and 92 articles in Chinese were included for full-text review. After full-text review, nine English and 29 Chinese articles were excluded because they did not report any information on measurement and survey administration. This resulted in a total of 28 articles in English and 63 articles in Chinese that were included in the final analysis. Among them, nine articles include two sub-studies each. One article includes three sub-studies. Therefore, 102 studies were included in the current analysis. A total of 81 studies focus on community health centers, 17 studies - on township health centers and four studies—on village clinics. The description of each study is provided in Supplement 2 and Supplement 3. 

### 2.3. Data Extraction and Analysis

A data extraction form in Excel was developed to collect the following information: author, published date, the year when the study was conducted, study setting, domains measuring PHC, items measuring PHC, response options and survey administration methods. Due to the variations in measurements and administration methods between studies, a descriptive reporting approach was used. 

## 3. Results

A total of 102 studies were included for data analysis. More than 80% (84) of the studies were published after 2009 when China launched its national health reform. Most studies were conducted in eastern (more developed) provinces in China. Guangdong (29), Shanghai (9) and Shandong (8) contributed to nearly half of the studies.

### 3.1. Patient Experience Measurements Adapted from Other Instruments

The measurements adapted from the instruments developed in other countries were used in over one-third (37 of 102) of the studies (Table 1). The PCAT and the WHO Health System Responsiveness Survey were the two instruments most often used in China. 

The original PCAT developed by Barbara Starfield at the Johns Hopkins Primary Care Policy Center is the most widely used instrument in China, including Guangdong [28,29,30,31,32,33,34,35,36,37,38], Shanghai [28,33,39], Hunan [40] and Tibet [41]. The original English PCAT adult version includes seven domains: First Contact (accessibility and utilization), Continuity, Coordination (information and referral systems), Comprehensiveness (service availability and service provided), Community Orientation, Family Centeredness and Cultural Competency. The response options are a four-point Likert-type response scale where 1 = definitely not, 2 = probably not, 3 = probably, 4 = definitely and an additional option 9 = not sure/do not know [24].

There are various Chinese versions of PCAT. Among 18 PCAT studies, 12 included all seven domains [29,33,34,35,36,37,38,41] and the remaining six used a subset [28,30,32,39,40,42]. For example, Community Orientation and Family Centeredness were not included in four studies [28,30,39,42] and Cultural Competency was not included in six studies [28,30,32,39,40,42]. Most studies followed the original four-point Likert-type response scale with the same wording but one study changed the wording of the response scale to 1 = never; 2 = sometimes; 3 = often; and 4 = always [30]. All PCAT studies included four of the seven domains (First Contact, Continuity, Coordination and Comprehensiveness), which correspond to Starfield’s “core domains” [24]. Although Comprehensiveness was included in all studies, the items in each study varied [24,40,43].

The Health System Responsiveness Survey was developed by the WHO to measure the users’ interaction with health services in eight domains: Dignity, Autonomy, Confidentiality, Communication, Prompt Attention, Quality of Basic Amenities, Social Support and Choice of health care providers. Questions measuring health system responsiveness cover ambulatory (22 items) and inpatient (11 items) visits. All questions used similarly ordered four-point (always, usually, sometimes, never) or five-point (mainly: very good, good, moderate, bad, very bad) Likert response options [23]. Among the 12 studies using this survey in China, only six studies included all eight domains [44,45,46,47,48,49]. Social Support was not included in five studies [50,51,52,53,54], Quality of Basic Amenities was not included in three studies [50,54,55] and Choice of Providers was not included in three studies [50,51,54]. Most studies used the response scale suggested by the WHO, with just two studies not doing so [48,49]. However, no studies reported the psychometric properties of measurements. 

### 3.2. Patient Experience Measurements Developed by Authors Themselves

Two-thirds (65 of 102) of the studies used measurements developed by the authors themselves. Most did not report the development process for these measurements. The frequency of PHC aspects, number of items and response options included in these measurements are summarized in Table 2 and Table 3. 

More than 40 care aspects were evaluated in the 65 studies. The care aspects most frequently evaluated, in decreasing order, were: service attitude of health professionals (92%); medical cost (82%); physical environment (78%); technical skill of health professionals (68%); and medical equipment (66%). Convenience and waiting times were evaluated in more than 50% of the studies. There are fewer studies that include interpersonal care aspects, such as privacy protection (25%), respect (12%), shared decision making (9%) and the doctor’s listening skills and patience (6%). 

The number of items reported in the studies ranged from 3 to 35, with the majority ranging from 5 to 15. A five-point-Likert response scale was used most often (50 out of 65) although some studies used three, four or six response options. The wording of five-point response options also varied. For example, some studies used “always, usually, sometimes, occasionally, never” or “completely agree, agree, not sure, disagree, completely disagree”. Other studies used “very good, good, neutral, bad, very bad” or “very satisfied, satisfied, neutral, dissatisfied, very dissatisfied”. Depending on the types of questions (declarative or evaluative) measuring patient perception, further research could explore appropriate evaluative language and response scales among Chinese people. 

Only six out of the 65 studies reported the psychometric properties of measurements [56,57,58,59,60,61]. Four studies reported good validity and reliability based on the results of Cronbach’s α and principle component analyses [56,57,58,61]. One study briefly reported good reliability and unacceptable validity without reporting any detail about data analysis [59]. One study only tested face and content validity through a pilot study with 30 respondents [60].

### 3.3. Survey Implementation Approach

The on-site survey was used by 99 studies while only three out of the 102 studies used a telephone survey. For the location of the on-site survey, the exit of primary care institutions is the most common place and was used by 63% of the studies. For the administration of the on-site survey, a face-to-face interview was reported by 29 studies and self-administration was reported by 14 studies, while this information was missing in the remaining 56 studies (Table 4). 

## 4. Discussion

Based on our previous research, we captured two major points relating to patient evaluation studies of PHC in China: the inconsistency of survey instruments and implementation approaches. Through a thorough discussion with PHC researchers in China and Canada, we agreed that a narrative review was an efficient approach to produce an overall picture about what measurements have been used and how they have been implemented among the patient evaluation studies of PHC in China. The advantage of this approach is that it offers breadth of literature coverage and the flexibility to deal with evolving knowledge and concepts, without requiring more time and resources to prepare and update the records as in a systematic review [62]. To include as many relevant publications as possible, we identified search terms in Chinese and English using a brainstorming technique within our research team, and developed the search strategy through an iterative approach (trying different wording of search terms and different combinations). Finally, we applied our search strategy in both PubMed and CNKI, which are the main databases that include publications about Chinese health services in English and Chinese, respectively. Although there is a small possibility that some relevant publications were missed in our search, we are confident that the results portray a comprehensive picture of patient evaluation studies of PHC in China. 

Through the analysis of 102 patient evaluation studies of PHC in China, we found that most studies used author-developed instruments with commonly used five-point Likert response options and many used Chinese translations of the instruments developed in other countries. Furthermore, most author-developed instruments measure the structural dimensions of PHC, and most measures focus on satisfaction to rate subjective perception rather than on care experience to report objective problems. 

### 4.1. Issues of Measurements Adapted from Other Countries

The studies included in our review used a similar approach to adapting instruments but the results varied even in the same context. This calls for national collaboration to address these issues. For example, three studies reported the adaptation process and validation results of PCAT in China [40,43,63]. Each used the following process: forward–backward translation; consensus by an expert panel to identify the appropriateness of items and domains; and a pilot test to improve item wording. However, the three Chinese versions vary, particularly in item wording. Even for the same item in the original PCAT, there were variations in some of the Chinese translations. These variations lead to varied findings of the psychometric properties of this instrument. For example, a validation study in Guangdong confirmed the construct factors embedded in the original PCAT [63]. However, another study in the same province using the same validation technique suggests that some domains should be removed, including Family Centeredness, Community Orientation and Cultural Competence as well as two items measuring First Contact and Coordination [43]. The psychometric analysis in the province of Hunan also showed that the factor constructs did not fit well with the underlying theoretical constructs in the original PCAT [40]. For each domain of primary care, First Contact fails to meet optimal psychometric standards of internal consistency in two studies in China. These studies found that Cronbach’s α was only 0.38 [43] and 0.48 [40] for First Contact-Utilization, respectively. The internal consistency of Comprehensiveness also showed inconsistent results between different studies [40,43,63]. 

The principles of First Contact, Continuity, Coordination and Comprehensiveness that are included in PCAT are recognized and shared internationally. However, service delivery and financing systems vary significantly between countries. The tools developed in one context may not be appropriate in another. For the same domain of primary care, the patients in different countries may have a different understanding and expectations. For example, First Contact items from the original PCAT may not be appropriate in countries with a different health system organization and patient expectations. Making appointments in advance and gatekeeping by primary care workers may not be applicable in some countries [64,65]. Geographical accessibility is a major constraint for local people to get to health care services in rural and remote areas, but it was not addressed in the original PCAT. Similar issues are likely to pertain to Comprehensiveness and Coordination with specialists. 

### 4.2. Issues of Measurements Developed by Authors

Most author-developed measures focus on the structural dimensions of PHC, such as the physical environment, medical equipment and convenience (distance from home to facility). The interest in predominantly evaluating the structural dimensions is based on the assumption that adequately trained doctors and nurses with access to infrastructure (such as well-equipped facilities and medicines) will be sufficient for guaranteeing adequate quality [66]. However, emerging evidence suggests that this understanding may be incorrect. For example, a cross-sectional study of 4,300 facilities in eight countries demonstrated that the structural input characteristics (amenities, equipment and medications) of health facilities are poorly correlated with the provision of evidence-based care (providers’ adherence to evidence-based care guidelines) [67]. Furthermore, if user perceptions in patient surveys closely reflect the observable infrastructural facilities, questions related to these perceptions would not add to what could be learned from direct observation using facility surveys [68]. These observations highlight the inappropriateness of evaluating the structural aspects of PHC from a user perspective. 

Although the technical skills of doctors are also often evaluated by patients in China, there is a weak correlation between the patient-perceived technical quality of care and technical quality measures extracted from medical records [69]. However, strong correlations were identified between the patient-perceived technical quality and their assessment of doctors’ interpersonal communication skills and trustworthiness [69]. Few patients have sufficient knowledge about their own illness or about possible treatment options to make an informed judgement about the technical quality of care [70]. When patients are asked about technical quality, they are more likely to make judgements based on the care aspects that they feel they are able to judge. Experts claim that clinician reports and medical chart reviews are better approaches to evaluating technical quality of care [71]. 

### 4.3. Satisfaction and Care Experience

Most patient survey measures of PHC in China focus on satisfaction to rate subjective perception rather than on patients’ care experience to report objective problems. In addition, there is increasing interest in patient satisfaction studies in hospital settings in China [71,72,73,74,75]. In developed countries, the rising consumerism in the 1970s and 1980s has led to an increased emphasis on patient perspectives [76]. The motivation for early patient surveys was to keep patients “happy” or “satisfied” rather than ask about what did or did not happen. Although extensive studies were conducted on patient satisfaction assessment, many of them did not explicitly elicit quality information from patients. Since the 1990s, it became clear that as a tool for quality improvement, the patient satisfaction survey was neither very sensitive nor very useful [76,77]. There is agreement that the definitive conceptualization of satisfaction with health care has still not been achieved and that understanding the process by which a patient becomes satisfied or dissatisfied remains unanswered [78]. Subsequently, patient surveys shifted towards reporting patient-reported care experience, which is now considered to provide more specific information to guide quality improvement compared to satisfaction measures [19,79,80,81]. The evolution of patient surveys from assessing satisfaction to focusing on care experience in developed countries suggests that apart from the increasing interest in patient satisfaction, patient care experience studies should also gain more attention in China. 

### 4.4. Survey Implementation

The patient exit survey in primary care institutions is the most commonly used survey implementation method in China. The popularity of the patient exit survey is due to its operational efficiency as it is more convenient to identify patients at clinics than through population-based surveys [82]. However, there are also arguments about the patient exit surveys that respondents in many cases do not openly report about negative experiences when they are within the premises of health facilities [68]. Another potential problem relates to the sampling approach. In almost all studies using the patient exit survey, the interviewer selected the next patient exiting the clinic after completing an interview. However, recent evidence shows that this sampling method yields a biased sample as patients who spend a longer time with the clinician are overrepresented. This bias can be removed by selecting the next patient who enters rather than exits the consultation room, which is operationally more efficient than alternative methods (systematic and simple random sampling) in most PHC settings [82]. In addition, most studies included in this review did not report clear information on sampling frames and patient recruitment procedures.

The two most popular survey administration methods in patient exit surveys in China are: face-to-face verbal interviews using paper and pencil; and the traditional paper and self-administration method involving handing paper questionnaires to people in person and asking them to complete them by hand and return them to the researcher. Some studies used both methods. Face-to-face interviews have the advantage of a high response rate and low cognitive burden for the respondents although this method leads to a high social desirability bias and interviewer bias [83]. The visual and written method of self-administration has the advantage of a low social desirability bias and high willingness to disclose sensitive information but places high cognitive burden on respondents, especially the demand for literacy [83]. Besides, the characteristics of these different survey administration modes may also influence participants’ response to the instruments, which needs to be examined further in the context of China.

## 5. Conclusions and Practice Implication

The patient experience measurements and survey implementation approaches are two major issues in patient evaluation studies of PHC in China. Most studies evaluate the structural dimensions of PHC, of which patients are not the best evaluators. Various wordings and categories of response options are used but it is unclear which one is optimal among Chinese people. Many studies do not report a detailed sampling approach or patient recruitment procedures and the effect of different survey administration methods on participants’ responses is unclear. 

The lack of consistency in survey instruments and administration methods indicates that more coordinated efforts are needed in order to develop valid instruments to evaluate patient perceptions of PHC performance in China. This study is only the first step to map out the current situation and propose future direction towards developing Chinese value-based patient experience measurements. As a starting point, explicit information about what matters most to patients in China should be investigated. Patient experience measurements should reflect patients’ needs and preferences in their cultural and health system contexts. Patient preferences or priorities have been studied extensively in Europe and North America. These efforts consistently demonstrate that in addition to clinical competency or technical quality, interpersonal care aspects, such as interpersonal communication, shared decision-making, respectfulness and relational continuity, are also very important for patients [84,85,86,87,88,89,90,91]. However, these health care aspects, their operational definitions and subsequent measures have all been developed in western countries with PHC-based health systems, which may not be applicable in other countries. Foundational work is needed to identify patient priorities in China through a strong collaboration among different stakeholders, especially with patients, caregivers and family members. This work could be performed through qualitative research using narrative-based analysis, followed by quantitative research using a discrete choice experiment. This work would result in consensus on which care aspects of PHC could be measured by patients.

Subsequently, the operational definitions and optimal response scales should be explored and identified. For each PHC care aspect prioritized by patients, we need to capture the measurable terms or phrases that they use when reporting their care experience. We also need to capture the evaluative language and categories that they use when they make judgement of “good” or “bad” health care. This type of work could generate the elements included in each PHC care aspect and the optimal response scale among Chinese patients, which will guide the selection of items and survey development. Based on these achievements, a research agenda of developing patient experience measurements could be envisioned. In general, this includes setting up a research team, selecting the domains and items, consensus process around finalizing the initial survey, pilot testing and refinement, examination of psychometric properties and knowledge translation. At the same time, a standardized survey implementation strategy could be developed, including determining the sample size for each institution, outlining the sampling approach, data collection approach, data entry and management, data analysis format and guidelines on data reporting. 

As China is moving towards building a people-centered integrated health system, measuring patient experience is an important approach to better understand patients’ needs and preferences and to ensure that people-centered care is delivered. Action plans are needed in order to promote the development of a patient experience measurement and the application of patient experience information. First, a national patient experience measurement strategy could be developed, which might be incorporated into the functions of the National Medical Service Management Center. This national strategy would aim to coordinate all efforts to measure patient experience, which would allow the government and health care providers to produce compelling data on which decisions can be based. The UK and the USA are world leaders in this field. Supervised by Care Quality Commission (CQC), England’s health care regulator, national patient experience surveys are conducted annually since 1998 through the National Health Service’s (NHS) Patient Survey Program. In the USA, patient experience surveys are mainly conducted through the Consumer Assessment of Healthcare Providers and Systems (CAHPS) program, which was established in 1995 by the Agency for Healthcare Research and Quality (AHRQ), the government agency responsible for health care quality and research. Recently, Canada has also initiated the process of developing a federal and provincial strategy for patient experience measurement. Similar work could be conducted in China through strong collaboration between health authorities, care providers and researchers, while considering the experiences from the above-mentioned countries. 

Second, more clarity is required around what information health authorities and health care providers need from patient experience surveys, including the format and how this information will be used by them in health policy making and quality improvement. International experiences have shown that managers and clinicians often struggle to link patient experience data to concrete transformations that could lead to an improved care experience [92]. These observations highlight the importance of involving local health authorities and health care providers in the development and implementation of patient experience surveys. Furthermore, exemplary primary care institutions with high patient experience performance should be selected as a benchmark in China. However, the link between organizational structure, management activities and patient experience performance is understudied. Further studies from leadership and change management perspectives at the health care organization level may deepen our understanding. 

Third, an effective public reporting program may also be established in order to inform patients of the care experience performance of different providers to guide their health care seeking behavior. Recognizing the value of transparency and accountability, publicly released performance information is not only viewed as a fundamental patient right but is also essential for improving the functioning of health care markets. There is evidence that consumers would use reports to support their decision making if the information was better designed and more relevant [93]. As China is expanding the family doctor contract service model, which aims to build a gatekeeper role of primary care clinics based on a long-term therapeutic relationship between family doctors and patients, publicly released performance information on family doctors may guide patients’ choice of their family doctor and facilitate the building of a longitudinal relationship. Based on the lessons learned from public reporting programs in other countries, such as low patient awareness of the existence of public reports, sub-optimal patient engagement in report development, information complexity and patients’ difficulty in processing numbers and abstract ideas, China could develop some innovative public reporting programs to target and deliver reports to Chinese patients.

## Figures and Tables

**Table 1:** Patient experience measurements of primary health care in the 102 studies.

Instrument	No. of Studies
Primary Care Assessment Tool (PCAT)	18
World Health Organization Health System Responsiveness Survey	12
Patient Assessment of Chronic Illness Care (PACIC)	2
Questionnaire of Continuity between Care Levels (CCAENA)	1
SERVQUAL	2
Australia patient satisfaction questionnaire	1
EUROPEP	1
Self-developed indicators by the authors	65
Total	102

**Table 2:** The top 20 most frequent evaluated primary health care aspects in the 65 studies with self-developed measurements.

Aspects of Primary Health Care	No. of Studies	Aspects of Primary Health Care	No. of Studies
Service attitude of health professionals	60	Treatment outcome	17
Medical charge and cost	53	Privacy protection	16
Physical environment	51	Procedures of visit	11
Technical skill of health professionals	44	Types of drugs	10
Medical equipment	43	Respect	8
Convenience	37	Length of consultation	8
Waiting time	36	Shared decision making	6
Doctors’ explanation	29	Whole person care	5
Overall rating	27	Appropriate treatment	5
Range of services	21	Trust	5

**Table 3:** Number of items and response scales in the 65 studies with self-developed measurements.

Number of Items	No. of Studies
<5	5
5–10	37
11–15	16
16–20	5
21–30	2
>30	1
**Response scales**	
5-point Likert scale	50
3-point Likert scale	5
4-point Likert scale	4
6-point Likert scale	3
Not reported	3

**Table 4:** Survey administration methods in the 102 studies.

Survey Method	No. of Studies
** On-site survey**	99
Place of survey	
Primary care institutions	64
Community or patient’ home	28
Not reported	7
Administration method	
Face-to-Face interview	29
Self-administration	14
Not reported	56
** Telephone survey**	3

## Supplementary Materials

The following are available online at https://www.mdpi.com/1660-4601/16/6/926/s1, Supplement 1: Search Strategy, Supplement 2: Patient evaluation studies published in English from PubMed, Supplement 3: Patient evaluation studies published in Chinese from China National Knowledge Infrastructure.
