# Supplementary material for: Evaluating Primary Health Care Performance from User Perspective in China: Review of Survey Instruments and Implementation Issues
Source: Int J Environ Res Public Health. 2019 Mar 14;16(6):926. doi: 10.3390/ijerph16060926 (PMC6466226; doi:10.3390/ijerph16060926)
Supplement: Supplementary file 1 [file ijerph-16-00926-s001.pdf]

**Article title**: Evaluating Primary Health Care Performance from User Perspective in China: Review of Survey Instruments and Implementation Issues

**Authors:** Wenhua Wang, Jeannie Haggerty, Ekaterina Loban, Xiaoyun Liu

**Corresponding author:** Xiaoyun Liu, China Center for Health Development Studies, Peking University

Email: xiaoyunliu@pku.edu.cn

**Supplement 1 Search Strategy**

**PubMed search were conducted on Feb 2018 using the following search strategy:**

(primary health care[Title] OR primary care[Title] OR community health[Title] OR township health center[Title/Abstract] OR township health centers[Title/Abstract] OR township hospitals[Title/Abstract] OR township hospital[Title/Abstract] OR village clinic[Title/Abstract] OR village clinics [Title/Abstract]) AND (china [Title] OR chinese[Title]) AND (evaluate[Title/Abstract] OR assess[Title/Abstract] OR assessment[Title/Abstract] OR evaluation[Title/Abstract] OR measure[Title/Abstract] OR measurement [Title/Abstract] OR survey [Title/Abstract] OR questionnaire[Title/Abstract] OR review[Title/Abstract] OR monitor[Title/Abstract]) AND (Patients[mesh] OR patient[tw] OR patients[tw] OR outpatient*[tw])

169 papers were identified.

**CNKI search were conducted on Feb 2018 using the following search strategy:**

文献总数：139 篇；检索条件：( ( 题名=社区卫生 并且 ( 摘要=病人 或者 摘要=患者 ) ) 并且 ( 主题=评估 或者 主题=评价 ) ) (精确匹配),专辑导航：全部; 数据库：文献 跨库检索

文献总数：117 篇；检索条件：( ( ( 关键词=村卫生室 或者 关键词=乡镇卫生院 ) 并且 ( 摘要=病人 或者 摘要=患者 ) ) 并且 ( 主题=评估 或者 主题=评价 ) ) (精确匹配),专辑导航：全部; 数据库：文献 跨库检索

文献总数：4 篇；检索条件：( ( ( 题名=乡镇卫生院 或者 题名=村卫生室 ) 并且 题名=居民 ) 并且 ( 主题=评价 或者 主题=评估 ) ) (精确匹配),专辑导航：全部; 数据库：文献 跨库检索

文献总数：42 篇；检索条件：( ( 题名=社区卫生 并且 题名=居民 ) 并且 ( 主题=评价 或者 主题=评估 ) ) (精确匹配),专辑导航：全部; 数据库：文献 跨库检索

**Supplement 2 Patient evaluation studies published in English from PubMed**

| Study | Study period | Region | Study setting | Sample size | Survey modality | Place of survey | Measurements |
|---|---|---|---|---|---|---|---|
| Kuang et al[1] | 2009 | Guangdong | CHC | 1645 | Face-to-face interview | On-site | PCAT |
| Di et al[2] | 2010 | Dalian | CHC | 372 | face-to-face interview | Not reported | 12 items with a 4-point scale |
| Chai et al[3] | 2011 | Chengdu | CHC | 1716 | Face to face | Household | 8 items with a five likert scale |
| Tang et al[4] | 2013 | Six provinces | CHC | 865 | Not reported | Not reported | 9 items with a 5-point Likert scale. |
| Tang et al[5] | 2013 | 18 provinces | CHC | 3306 | face-to-face interview | Household | 9 items with a 5-point Likert scale |
| Wang et al[6] | 2013 | Guangdong | CHC | 1440 | Face-to-face interview | On-site | PCAT |
| Yang[7] | 2013 | Hunan | CHC | 2532 | Face-to-face interview | On-site | PCAT |
| Liu et al[8] | 2013 | Shanghai | CHC | 960 | Not reported | Household | PACIC |
| Li et al.[9] | 2014 | Shenzhen | CHC | 787 | face-to-face interview | On-site | 13 items with a 5-point Likert scale |
| Dong et al [10] | 2014 | 35 cities | CHC | 88482 | Not reported | On-site | 12 items with a 5-point Likert scale |
| Wei et al[11] | 2015 | Shenzhen Shanghai | CHC | 2721 | Face-to-face interview | On-site | PCAT |
| Wang et al[12] | 2015 | Tibet | THC | 1386 | Face-to-face interview | On-site | PCAT |
| Shi et al[13] | 2015 | Guangdong | CHC | 560 | Face-to-face interview | On-site | PCAT |
| Du et al[14] | 2015 | Guangdong | CHC THC | 864 | Face-to-face interview | On-site | PCAT |
| Li et al[15] | 2015 | SH SZ | CHC | 568 128 | Face-to-face interview | On-site | PCAT |
| Zeng et al[16] | 2015 | Guangdong | CHC THC VC | 736 | Face-to-face interview | Household | PCAT |
| Yin et al[17] | 2015 | national | CHC | 12386 | face-to-face interview | On-site | 9 items with a 5-point Likert scale |
| Sun et al[18] | 2015 | Shanghai | CHC | Not reported | Not reported | Not reported | 10 items a 5-point Likert scale |
| Hu et al[19] | 2016 | Guangdong | CHC THC | 864 | Not reported | On-site | PCAT |
| Li et al[20] | 2016 | Jilin | CHC THC | 993 | Face-to-face interview | Household | 2 items with a 3-point Likert scale, and o5 items with a 5-point Likert scale. |
| Wu et al[21] | 2016 | Shenzhen | CHC | 3848 | Not reported | On-site | 8 items a 5-point Likert scale |
| Li et al[22] | 2017 | Jiangsu | CHC | 1058 | Not reported | On-site | EUROPEP with a 5-point Likert-type scale |

| | | | | | | | |
|---|---|---|---|---|---|---|---|
| Liu et al[23] | 2017 | Beijing | CHC | 700 | Face-to-face interview | On-site | Questionnaire of Continuity between Care Levels with a five-point Likert scale. |
| Wang et al[24] | 2017 | Eight provinces | THC | 3435 | Face-to-face interview | Household | WHO Health System Responsiveness Survey |
| Wei et al[25] | 2017 | Shenzhen Shanghai Hongkong | CHC | 797 802 1325 | Face-to-face interview Telephone survey for HK | On-site | PCAT |
| Feng et al[26] | 2017 | Guangdong | VC | 1491 | Face-to-face interview | Household | PCAT |
| Wu et al [27] | 2017 | Zhejiang | CHC | 1248 | Not reported | Not reported | 7 items with a 4-point Likert scales |
| Li et al[28] | 2017 | Shenzhen | CHC | 6887 | Intercept interview | On-site | 6 items a 5-point Likert scale |

Note: CHC: Community Health Center; THC: Township Health Center; VC: Village Clinic; PCAT: Primary Care Assessment Tool; PACIC: Patient Assessment of Chronic Illness Care; WHO: World Health Organization.

**Reference**


1. Kuang L, Liang Y, Mei J, et al. Family practice and the quality of primary care: a study of Chinese patients in Guangdong Province. *Family practice* 2015; **32**(5): 557-63.
2. Dib HH, Sun P, Minmin Z, Wei S, Li L. Evaluating community health centers in the City of Dalian, China: how satisfied are patients with the medical services provided and their health professionals? *Health & place* 2010; **16**(3): 477-88.
3. Chai Y, Xu H, Wang W, et al. A survey of factors associated with the utilization of community health centers for managing hypertensive patients in Chengdu, China. *PloS one* 2011; **6**(7): e21718.
4. Tang C, Luo Z, Fang P, Zhang F. Do patients choose community health services (CHS) for first treatment in China? Results from a community health survey in urban areas. *Journal of community health* 2013; **38**(5): 864-72.
5. Tang L. The Chinese community patient's life satisfaction, assessment of community medical service, and trust in community health delivery system. *Health and quality of life outcomes* 2013; **11**: 18.
6. Wang HH, Wong SY, Wong MC, et al. Patients' experiences in different models of community health centers in southern China. *Annals of family medicine* 2013; **11**(6): 517-26.
7. Yang H, Shi L, Lebrun LA, Zhou X, Liu J, Wang H. Development of the Chinese primary care assessment tool: data quality and measurement properties. *International journal for quality in health care : journal of the International Society for Quality in Health Care* 2013; **25**(1): 92-105.
8. Liu LJ, Li Y, Sha K, Wang Y, He X. Patient assessment of chronic illness care, glycemic control and the utilization of community health care among the patients with type 2 diabetes in Shanghai, China. *PloS one* 2013; **8**(9): e73010.
9. Li H, Chung RY, Wei X, et al. Comparison of perceived quality amongst migrant and local patients using primary health care delivered by community health centres in Shenzhen, China. *BMC family practice* 2014; **15**: 76.
10. Dong X, Liu L, Cao S, et al. Focus on vulnerable populations and promoting equity in health service utilization--an analysis of visitor characteristics and service utilization of the Chinese community health service. *BMC public health* 2014; **14**: 503.
11. Wei X, Li H, Yang N, et al. Changes in the perceived quality of primary care in Shanghai and Shenzhen, China: a difference-in-difference analysis. *Bulletin of the World Health Organization* 2015; **93**(6): 407-16.
12. Wang W, Shi L, Yin A, et al. Primary care quality among different health care structures in Tibet, China. *BioMed research international* 2015; **2015**: 206709.

13. Shi L, Lee DC, Liang H, et al. Community health centers and primary care access and quality for chronically-ill patients - a case-comparison study of urban Guangdong Province, China. *International journal for equity in health* 2015; **14**: 90.
14. Du Z, Liao Y, Chen CC, Hao Y, Hu R. Usual source of care and the quality of primary care: a survey of patients in Guangdong province, China. *International journal for equity in health* 2015; **14**: 60.
15. Li H, Wei X, Wong MC, Wong SY, Yang N, Griffiths SM. A Cross-Sectional Comparison of Perceived Quality of Primary Care by Hypertensive Patients in Shanghai and Shenzhen, China. *Medicine* 2015; **94**(34): e1388.
16. Zeng J, Shi L, Zou X, Chen W, Ling L. Rural-to-Urban Migrants' Experiences with Primary Care under Different Types of Medical Institutions in Guangzhou, China. *PloS one* 2015; **10**(10): e0140922.
17. Yin X, Gong Y, Yang C, et al. A Comparison of Quality of Community Health Services Between Public and Private Community Health Centers in Urban China. *Medical care* 2015; **53**(10): 888-93.
18. Sun X, Li Y, Liu S, et al. Enhanced Performance of Community Health Service Centers during Medical Reforms in Pudong New District of Shanghai, China: A Longitudinal Survey. *PloS one* 2015; **10**(5): e0125469.
19. Hu R, Liao Y, Du Z, Hao Y, Liang H, Shi L. Types of health care facilities and the quality of primary care: a study of characteristics and experiences of Chinese patients in Guangdong Province, China. *BMC health services research* 2016; **16**(a): 335.
20. Li J, Wang P, Kong X, Liang H, Zhang X, Shi L. Patient satisfaction between primary care providers and hospitals: a cross-sectional survey in Jilin province, China. *International journal for quality in health care : journal of the International Society for Quality in Health Care* 2016; **28**(3): 346-54.
21. Wu J, Zhang S, Chen H, et al. Patient Satisfaction with Community Health Service Centers as Gatekeepers and the Influencing Factors: A Cross-Sectional Study in Shenzhen, China. *PloS one* 2016; **11**(8): e0161683.
22. Li WZ, Gan Y, Zhou YF, et al. Factors Affecting Patient Satisfaction with Community Health Service under the Gatekeeper System: A Cross-sectional Study in Nanjing, China. *Biomedical and environmental sciences : BES* 2017; **30**(9): 685-90.
23. Liu C, Wu Y, Chi X. Relationship preferences and experience of primary care patients in continuity of care: a case study in Beijing, China. *BMC health services research* 2017; **17**(1): 585.
24. Wang W, Maitland E, Nicholas S, Loban E, Haggerty J. Comparison of patient perceived primary care quality in public clinics, public hospitals and private clinics in rural China. *International journal for equity in health* 2017; **16**(1): 176.
25. Wei X, Yin J, Wong SY, Griffiths SM, Zou G, Shi L. Private ownership of primary care providers associated with patient perceived quality of care: A comparative cross-sectional survey in three big Chinese cities. *Medicine* 2017; **96**(1): e5755.
26. Feng S, Shi L, Zeng J, Chen W, Ling L. Comparison of Primary Care Experiences in Village Clinics with Different Ownership Models in Guangdong Province, China. *PloS one* 2017; **12**(1): e0169241.
27. Wu D, Lam TP, Lam KF, Zhou XD, Sun KS. Public views towards community health and hospital-based outpatient services and their utilisation in Zhejiang, China: a mixed methods study. *BMJ open* 2017; **7**(11): e017611.
28. Li W, Gan Y, Dong X, et al. Gatekeeping and the utilization of community health services in Shenzhen, China: A cross-sectional study. *Medicine* 2017; **96**(38): e7719.

**Supplement 3 Patient evaluation studies published in Chinese from China National Knowledge Infrastructure**

| Study | Study period | Region | Study setting | Sample size | Survey modality | Place of survey | Measurements |
|---|---|---|---|---|---|---|---|
| 1 Wang et al | 2017 | Changchun | CHC | 430 | Face-to-face interview | On-site | SERVQUAL |
| 2 Yu et al | 2017 | Zunyi | THC | 150 | NR | On-site | SERVQUAL 22 items with 5-point Likert scale satisfaction |
| 3 Lin et al | 2017 | Shenzhen | CHC | 726 | Household | NR | PCAT |
| 4 Pu et al | 2017 | Chongqing | VC | 858 | NR | Household | 13 items with 3-point Likert scale good to bad |
| 5 Liu et al | 2017 | Jiangsu | THC | 3930 | Face-to-face interview | Household | 7 items with 5-point Likert scale bad to good |
| 6 Zhang | 2017 | Chongqing | CHC | 196 | Self administered | On-site | 4 items with 5-point Likert scale satisfaction |
| 7 Liu et al | 2016 | Shenzhen | CHC | 476 | Self administered | On-site | PCAT |
| 8 Chen et al | 2015 | Zunyi | THC | 144 | NR | On-site | 4 domains 14 items with 5-point Likert scale satisfaction |
| 9 He et al. | 2015 | Henan | CHC | 240 | NR | NR | 5 domains 18 items with 5-point Likert scale satisfaction |
| 10 Peng et al. | 2015 | Chongqing | CHC | 402 | NR | On-site and Household | 7 items with 5-point Likert scale bad to good |
| 11 Wu et al | 2015 | Henan | CHC | Not clear | Self administered | Household | WHO Health System Responsiveness Survey |
| 12 Xiang | 2015 | 4 provinces | THC | 1054 | NR | Household | 3 items with 4-point Likert scale bad to good |
| 13 Tang | 2015 | Chengdu | CHC | 490 | Self administered | NR | 4 domains 9 items with 5-point Likert scale satisfaction |
| 14 Deng | 2015 | Chongqing | THC | 393 | NR | On-site | 14 items with 5-point Likert scale satisfaction |
| 15 Si | 2015 | Shandong | CHC | 421 | NR | On-site | 5 domains 29 items with 5-point Likert scale satisfaction |
| 16 Dong et al | 2015 | Yun nan | THC | 257 | Face-to-face interview<br>Self administered | On-site | 10 items with 5-point Likert scale satisfaction plus hard to say |
| 17 Zhang | 2014 | Jiangsu | THC | 200 | NR | Household | 15 items with 5-point Likert scale satisfaction |
| 18 Yu et al | 2014 | Guangdong | CHC | 2507 | NR | On-site | 15 items with 5-point Likert scale satisfaction |

| | | | | | | | |
|---|---|---|---|---|---|---|---|
| 19 Zhang et al | 2013 | Guangdong | CHC | 198 | NR | On-site | 6 domains 23 items with 5-point Likert scale satisfaction |
| 20 Han et al | 2013 | Shandong | CHC | 476 | NR | Household | 4 domains 11 items with 5-point Likert scale satisfaction |
| 21 Yao et al | 2013 | Xinjiang | CHC | 600 | Self administered | On-site | 10 items with 5-point Likert scale satisfaction |
| 22 Wu et al | 2013 | Guangdong | CHC | 720 | NR | On-site | 10 items with 5-point Likert scale satisfaction |
| 22 Wu et al | 2013 | Guangdong | CHC | 116 | telephone | | 5 items with 5-point Likert scale satisfaction |
| 23 Liu et al | 2013 | Shanghai | CHC | 900 | Self administered | On-site | 5 domains 11 items with 5-point Likert scale satisfaction |
| 24 Wang et al | 2013 | Hubei | CHC | 298 | Self administered | On-site | 10 items with 5-point Likert scale satisfaction |
| 25 Wu et al | 2013 | Liaoning | CHC | 400 | NR | On-site | 6 items with 5-point Likert scale satisfaction |
| 26 Yang | 2013 | Jiangxi | CHC | 3023 | NR | On-site and Household | 10 items with 5-point Likert scale satisfaction |
| 27 Yang et al | 2012 | Qingdao | CHC | 332 | NR | On-site | 5 items with 5-point Likert scale satisfaction |
| 28 Gong | 2013 | Shandong | CHC | 299 | Not clear | On-site | PACIC |
| 29 Huang | 2012 | Guangzhou | CHC | 2992 | Face-to-face interview and Self administered | On-site and Household | 4 domains 20 items with 5-point Likert scale plus don't know option satisfaction |
| 30 Li | 2012 | Nanchang | CHC | 503 | NR | On-site | 9 items nc |
| 31 Han | 2011 | Shandong | VC | 2226 | Face-to-face interview | Household | 5 items with 3,4,5 response options |
| 31 Han | 2011 | Shandong | THC | 2226 | Face-to-face interview | Household | 5 items with 5-point Likert scale bad to good or 3-response options |
| 32 Ge | 2011 | 3 provinces | THC | 1147 | NR | On-site | 9 items with 5-point Likert scale satisfaction plus not clear |
| 33 Wang | 2011 | Anhui | CHC | 1200 | Face-to-face interview | On-site | 8 items with 5-point Likert scale satisfaction |
| 34 Qiu et al | 2010 | Shandong | CHC | 576 | Face-to-face interview | Household | 2 domains 15 items with 3-point Likert scale satisfaction |
| 35 Xiao | 2010 | Zhejiang | CHC | 250 | NR | On-site | 6 items with 4-point Likert scale satisfaction |

| | | | | | | | |
|---|---|---|---|---|---|---|---|
| 36 Liang | 2010 | Guangxi | CHC | 436 | telephone | | 10 items with 3-point Likert scale satisfaction |
| 37 Yuan et al | 2009 | Shanxi | CHC | 460 | Face-to-face interview | On-site | 8 domains 35 items with 5-point Likert scale agree |
| 38 Tang | 2011 | Shanghai | CHC | 934 | Face-to-face interview | On-site | 18 items with 5-point Likert scale bad to good |
| 39 Jiang et al | 2009 | Jiangxi | CHC | 500 | Self administered | On-site | 8 items not clear |
| 40 Sun et al | 2009 | Hubei | CHC | 165 | NR | On-site | 6 domains 17 items with 5-point Likert scale satisfaction |
| 41 Xiao | 2009 | Shanghai | CHC | 1623 | NR | On-site | 4 domains 14 items with 5-point Likert scale satisfaction |
| 42 Pan et al | 2009 | Fujian | CHC | 706 | NR | Household | 10 items with 5-point Likert scale satisfaction |
| 42 Pan et al | 2009 | Fujian | CHC | 706 | NR | Household | WHO Health System Responsiveness Survey |
| 43 Duan | 2009 | Sichuan | CHC | 247 | NR | On-site | 12 items with 5-point Likert scale satisfaction |
| 44 Ma | 2009 | Zhejiang | CHC | Not clear | NR | On-site | 8 items with 5-point Likert scale satisfaction |
| 45 Zhu et al | 2009 | Jiangsu | CHC | 300 | Self administered | On-site | WHO Health System Responsiveness Survey |
| 46 Chou | 2009 | Shanghai | CHC | 984 | NR | Household | 13 items with 5-point Likert scale satisfaction |
| 47 Lin et al | 2009 | Heilongjiang | CHC | 660 | NR | On-site | 8 items nc |
| 48 Ma et al | 2009 | Shandong | CHC | 692 | face-to-face interview | On-site | WHO Health System Responsiveness Survey |
| 49 Wang et al | 2008 | Fujian | CHC | 162 | face-to-face interview | On-site | WHO Health System Responsiveness Survey |
| 50 Xiong | 2008 | 3 provinces | THC | | NR | Household | 4 items with 3-point Likert scale good to bad |
| 51 Wang | 2008 | Hubei | CHC | NR | NR | On-site Household | WHO Health System Responsiveness Survey |
| 51 Wang | 2008 | Hubei | CHC | Not clear | NR | On-site and Household | 4 items with 5-point Likert scale bad to good |
| 52 Cao | 2008 | Liaoning | CHC | 697 | NR | On-site | 6 items with 5-point Likert scale satisfaction |
| 53 Hao | 2007 | 10 provinces | CHC | 1168 | NR | On-site | 18 items with 5-point Likert scale satisfaction |

| | | | | | | | |
|---|---|---|---|---|---|---|---|
| 54 Pan et al | 2007 | Fujian | CHC | 188 | NR | On-site and Household | 7 items with 5-point Likert scale satisfaction |
| 55 Colette et al | 2007 | Beijing | CHC | 700 | Self administered | On-site | Australia patient satisfaction questionnaire |
| 56 Wang | 2007 | Guangdong | CHC | 848 | Face-to-face interview | On-site | 12 items with 5-point Likert scale satisfaction |
| 56 Wang | 2007 | Guangdong | CHC | 755 | NR | Household | WHO Health System Responsiveness Survey |
| 57 Liu et al | 2007 | Hubei | CHC | 99 | NR | On-site | WHO Health System Responsiveness Survey |
| 58 Zhang et al | 2007 | Shanghai | CHC | 502 | NR | NR | WHO Health System Responsiveness Survey |
| 59 Xie et al | 2005 | Guangdong | CHC | 825 | NR | NR | 7 items with 5-point Likert scale satisfaction plus not clear |
| 60 Li et al | 2005 | Guangdong | CHC | 832 | NR | On-site | WHO Health System Responsiveness Survey |
| 61 Mao | 2004 | Zhejiang | CHC | Not clear | Not clear | On-site telephone | 3 items with 5-point Likert scale bad to good |
| 62 Zhu | 2004 | NR | THC | 70 | NR | On-site | 10 items with 5-point Likert scale satisfaction |
| 63 Yin | 2004 | Shanghai | CHC | 780 | NR | On-site | WHO Health System Responsiveness Survey |
| 63 Yin | 2004 | Shanghai | CHC | 780 | NR | On-site | 11 items with 5-point Likert scale satisfaction |

Note: CHC: Community Health Center; THC: Township Health Center; VC: Village Clinic; PCAT: Primary Care Assessment Tool; PACIC: Patient Assessment of Chronic Illness Care; WHO: World Health Organization; NR: Not reported.

**Reference list**


1. 王竞,李晶华,张莉,马天娇,孔璇,岳晓文.长春市不同举办主体社区卫生服务中心患者感知服务质量评价[J].医学与社会,2017,30(12):15-17+28.
2. 于金娜,向越云.运用 SERVQUAL 量表评价乡镇卫生院医疗服务质量[J].陕西广播电视大学学报,2017,19(03):83-86.
3. 林滢宇,张升超,余信国,张翠芬.基于 PCAT 量表分析社区卫生服务质量对高血压患者管理效果的影响[J].中国社会医学杂志,2017,34(04):369-371.
4. 蒲鑫鑫,龙华,何中臣,唐贵忠.农村居民对村卫生室的利用及评价调查研究——以重庆市 5 个区县为例[J].中国卫生事业管理,2017,34(05):370-372.
5. 刘雪仪. 农村慢性病服务纵向整合现状研究[D].南京医科大学,2017.
6. 张力. 重庆市社区卫生服务中心实施绩效管理对基本医疗服务的影响研究[D].重庆医科大学,2017.
7. 刘晓,高青,游寒剑,付敏,于露怡,梁希,陈少贤.举办医院的等级对深圳市社区卫生服务机构基本医疗服务质量的影响[J].医学与社会,2016,29(01):17-19+26.

8. 陈艾艾,蒋位哲,刘仕方,强威,段自坤.遵义县乡镇医院患者满意度调查与分析[J].辽宁医学院学报(社会科学版),2015,13(04):57-60.
9. 贺睿博,李军山,殷晓露,刘秋旭.基于模糊综合评价法的河南省社区卫生服务居民满意度研究[J].中国全科医学,2015,18(16):1901-1904.
10. 彭韵杨,伍林生.重庆市社区居民对社区卫生服务的情感倾向研究[J].中国全科医学,2015,18(16):1909-1913.
11. 吴辉,李玉春,蔺琳,丁宇,吴卫东.关键知情人对河南省社区卫生服务机构反应性的评价研究[J].中国卫生政策研究,2015,8(05):48-53.
12. 项远兮. 基于乡村一体化管理政策的农村卫生服务资源整合研究[D].华中科技大学,2015.
13. 唐峻炜. 龙泉驿区城市社区卫生服务居民满意度研究[D].南华大学,2015.
14. 邓梦露. 重庆市乡镇卫生院绩效管理实施现状及影响因素分析[D].重庆医科大学,2015.
15. 司明舒. 城市社区卫生服务居民满意度调查研究[D].河北经贸大学,2015.
16. 董安娜,李燕,李伟明,张京晶,阮真真,毛颖,段如菲.云南乡镇卫生院门诊患者满意度及其影响因素分析[J].卫生软科学,2014,28(05):268-271.
17. 张思冬. 乡镇卫生院绩效现状与问题研究[D].南京医科大学,2014.
18. 余雪强,周少维,黄翔,赖秀娟,杨华杰,周志衡,王家骥.中山市社区居民对社区卫生服务的满意度及其影响因素分析[J].中国初级卫生保健,2014,28(01):41-43.
19. 张立威,黄婉霞,王家骥.应用模糊综合评判法评价社区卫生服务患者满意度[J].中国卫生事业管理,2013,30(12):890-892.
20. 韩春蕾,郝冰冰.新医改形势下烟台市社区卫生服务患者满意度调查分析[J].中国卫生统计,2013,30(05):644-646.
21. 姚萱,杨红,周天虹,买买提·牙森,张伟,邓峰.乌鲁木齐市居民社区卫生服务满意度调查与评价[J].新疆医科大学学报,2013,36(10):1526-1529+1534.
22. 巫云辉,郝晓宁,邱德星,李惠霞,梁丽娟,程学添,王贤初,麦艳冰.深圳市光明新区社区卫生服务绩效管理效果评价研究[J].中国全科医学,2013,16(34):3304-3307.
23. 刘元凤,荆丽梅,娄继权,丁晔.浦东新区社区卫生服务中心患者满意度调查分析[J].卫生经济研究,2013(09):20-22.
24. 王留明,喻娟.武汉市某区社区卫生服务患者满意度及影响因素因子分析[J].医学与社会,2013,26(06):32-34.
25. 伍芳,何钦成,马亚楠,郭锋,郭红梅.沈阳市郊区居民社区卫生服务满意度调查[J].现代预防医学,2013,40(10):1874-1876+1879.
26. 杨晓金. 九江市城市社区卫生服务机构卫生服务能力及效果评价的研究[D].南昌大学,2013.
27. 杨文文,曹勇,张云,郑媚.青岛市社区卫生服务机构居民满意度调查分析[J].卫生软科学,2012,26(10):861-863.
28. 宫晓. 基于患者评价的山东省三市社区卫生机构慢性病管理现状研究[D].山东大学,2012.
29. 黄玮. 广州市居民社区卫生服务满意度调查研究[D].广东药学院,2012.
30. 李钟捷. 南昌市社区卫生服务现状及综合评价研究[D].南昌大学,2012.
31. 韩虎. 山东省农村居民对不同级别农村医疗机构就医状况评价研究[D].山东大学,2011.
32. 葛智馨. 我国部分农村地区乡村两级卫生机构卫生服务质量现状分析和指标体系设计研究[D].复旦大学,2011.
33. 王法艳. 合肥市社区卫生服务体系建设效果评价研究[D].安徽医科大学,2011.
34. 邱瑞香,王汝芬,赵林飞.潍坊市中心城区居民对社区卫生服务现状的评价调查报告[J].中国初级卫生保健,2010,24(10):31-33.
35. 肖娟.杭州社区卫生服务患者满意度与影响因素研究[J].现代物业(中旬刊),2010,9(09):87-88.
36. 梁耀元. 社区卫生服务居民满意度评价指标赋权方法的探讨[D].广西医科大学,2010.
37. 原静,刘桂芬,王萍,孟保平,李秀丽.太原市社区卫生服务居民满意度调查与评价[J].中国药物与临床,2009,9(12):1184-1186.
38. 汤真. 上海市闸北区社区卫生服务提供状况与评价[D].复旦大学,2009.
39. 江泽慧,周小军,钟豪翔.南昌市居民对社区卫生服务利用和满意度评价[J].中国卫生资源,2009,12(06):292+294.

40. 孙树学,范翠萍,龚勋,徐俊,金威,刘燕,姚岚.武汉市社区卫生服务居民满意度评价指标体系实证研究[J].医学与社会,2009,22(11):20-21.
41. 肖霞. 上海市社区卫生服务质量改进与发展策略研究[D].复旦大学,2009.
42. 潘志明,郑振佺,刘永前.福州市居民对社区卫生服务的满意度与反应性评价[J].中国全科医学,2009,12(17):1652-1653+1656.
43. 段桂敏.成都市社区卫生服务患者满意度影响因素分析[J].社区医学杂志,2009,7(16):1-4.
44. 马海燕.社区卫生服务满意度快速评价研究[J].卫生软科学,2009,23(04):372-375.
45. 朱奇,王青龙,王友书,曹红梅,陈翠丽.南通市区社区卫生服务反应性调查与分析[J].南通医学院学报,2009,29(04):259-262.
46. 仇育彬. 员工工作满意度、居民满意度及其与社区卫生服务发展的关系[D].复旦大学,2010.
47. 林宏琴,王东雷,刘英,李世娥.哈尔滨市城市居民利用社区卫生服务现状与满意度评价[J].中国社区医师(医学专业半月刊),2009,11(09):185-186.
48. 马起龙,尹文强,黄冬梅,孟梦,李丹.山东省三城市社区卫生服务机构反应性调查[J].中国初级卫生保健,2009,23(04):26-28.
49. 汪雪莲,郑振佺,王红连,江巧瑜.福州市社区卫生服务反应性的模糊综合评价[J].中国初级卫生保健,2008(11):18-20.
50. 熊巨洋. 农村地区乡镇卫生院绩效评价研究[D].华中科技大学,2008.
51. 汪云. 区级社区卫生服务系统绩效评价指标体系研究[D].华中科技大学,2008.
52. 曹颖. 沈阳市城郊四区社区卫生服务的需求及其影响因素研究[D].中国医科大学,2008.
53. 郝晓宁. 中国城市社区卫生服务运行机制与制度建设研究[D].山东大学,2007.
54. 潘志明,郑振佺,刘贤忠,曾美玲.福州市居民对社区卫生服务评价调查分析[J].社区医学杂志,2007(21):16-17.
55. Colette Browning,Shane Thomas,杨辉,张拓红,李小飞,任侬,李志新.社区老年病人怎样评价社区卫生服务的质量[J].中国全科医学,2007(21):1757-1760.
56. 汪胜. 深圳市公明街道社区卫生服务需求与利用研究[D].昆明医学院,2007.
57. 刘冰,高向华,王芳,贾利高,严彬,卢祖洵.十堰市社区卫生服务反应性综合评价[J].中国初级卫生保健,2007(04):33-35.
58. 张青鸟,姚有华,李琛.上海市闸北区社区卫生服务反应性评价[J].中国初级卫生保健,2007(01):28-30.
59. 解瑞谦,刘军安,孙奕,卢祖洵.深圳市居民社区卫生服务满意度及排序评价[J].中国全科医学,2005(07):544-546.
60. 杨德华,李谨邑,孙奕,卢祖洵.深圳市社区卫生服务反应性测量及影响因素分析[J].中国全科医学,2005(05):359-362.
61. 毛一萍. 杭州市社区卫生服务现状及趋势发展研究[D].浙江大学,2004.
62. 诸爱图. 丁桥镇农村社区卫生服务改革研究[D].浙江大学,2004.
63. 尹文强. 城市社区卫生服务现状评价与可持续发展策略研究[D].复旦大学,2003.
